# “Stop, don’t touch, run away!”: reconceptualizing firearm industry-funded youth education programs as corporate political activity

**DOI:** 10.1186/s12992-025-01106-7

**Published:** 2025-05-09

**Authors:** May C. I. van Schalkwyk, Benjamin Hawkins, Nason Maani, Mark Petticrew

**Affiliations:** 1 Global Health Policy Unit, School of Social and Political Science, University of Edinburgh, Edinburgh, EH8 9LD UK; 2 Centre for Pesticide Suicide Prevention, University of Edinburgh, Edinburgh, EH16 4TJ UK; 3 MRC Epidemiology Unit, University of Cambridge, Addenbrookes Hospital, Cambridge, CB2 0QQ UK; 4 Faculty of Public Health and Policy, London School of Hygiene and Tropical Medicine, 15-17 Tavistock Place London, London, WC1H 9SH UK

**Keywords:** Child health policy, Firearm injuries, Firearm industry, Commercial determinants of health, Gun safety education, Corporate political activity

## Abstract

**Background:**

Injuries represent a major threat to child health globally. In the US, firearm injuries are the leading cause of death among children and adolescents. Despite limited evidence of their effectiveness industry-funded bodies promote the delivery of their youth education programs while lobbying against firearm control policies. This article analyzes how the National Rifle Association (NRA) frames issues of gun ownership, safety and the role of the Eddie Eagle GunSafe^®^ program as an effective firearm safety intervention and examines how the design, promotion and delivery of the program serves the corporate political interests of the firearm industry at the expense of public health.

**Methods:**

We conducted an analysis of Eddie Eagle Gunsafe^®^ program-related materials and the NRA’s practices to promote the program’s legitimacy and effectiveness, by applying published taxonomies of corporate framing and action strategies. Data were collected from the program-specific websites and other NRA outlets to capture the breadth of strategies used by the NRA.

**Results:**

The NRA’s education-related practices support the firearm industry’s political agenda. The NRA adopts framing and action strategies that present the presence of firearms in homes and communities as inevitable and normal, and the education of children through the delivery of their “lifesaving” program as the common-sense and effective way of keeping children safe from firearm injuries. They make misleading claims about the effectiveness of the Eddie Eagle Gunsafe^®^ program while undermining the credibility of those who advocate for child safety, including mothers and public health actors.

**Conclusion:**

The delivery of the Eddie Eagle GunSafe^®^ program needs critical scrutiny as is increasingly applied to other industry-funded initiatives. Policies based on a recognition that children and adolescents are safest when their homes and communities are free of firearms are needed. Findings from this analysis are relevant beyond the US and can be used to inform the governance of child safety and injury prevention globally. Analysis of the firearm industry extends the literature on the commercial determinants of health to an important new sector with significant impacts on global health.

‘*Our goal is keeping children safe and parents informed… We want to provide Eddie Eagle’s message and safety materials to every family we can’*

Eric Lipp, National Community Outreach Department Manager, NRA 2019 [1]

## Background

Globally, injuries represent a major driver of death and disability among children and young people, with three of the top five causes of death among those aged 5–29 years being injury related [2]. Furthermore, the burden of injury-related morbidity and mortality is inequitably distributed within and between countries, with children from among disadvantaged communities experiencing the greatest risk irrespective of country income level [2]. The prevention of child injuries is of key importance in realizing the UN Sustainable Development Goals but has received relatively little attention from scholars of global health and the commercial determinants of health (CDOH), despite a substantial body of evidence on the effectiveness of injury prevention measures [2, 3]. Greater understanding is needed of the factors that undermine progress in addressing the significant impact injuries have on children’s health and life opportunities, including the role of vested interests and lack of governance structures. A particularly under-studied issue in this context are the harms resulting from firearm injuries and the role of the firearm industry in harm prevention debates [4]. Given both the proliferation of firearms and the key political importance of the firearm lobby, the US represents an important and illuminating case study for understanding the role of the firearm industry as a CDOH [5]. However, the findings here will be of relevance also to gun control and injury prevention debates in other contexts as well as building on the wider literature on commercial actors as a key determinant of global health.

In the US, firearm injuries are the leading cause of death among children and adolescents aged 1 to 19 years, eclipsing deaths caused by road traffic injuries since 2020 [6]. The US has one of the highest population level firearm mortality rates, and within specific sub-populations such as women, adolescents and children, both globally and among high income countries [7]. Indeed, US death rates due to firearm violence are more comparable to those observed in countries affected by active conflict [7]. In 2015, 98.1% of all children killed by firearms in high income countries were in the US [8]. Beyond mortality, firearm injuries are associated with greater injury severity and health care utilization than other forms of penetrating trauma, [9] and there is emerging evidence on the negative mental health sequelae of pediatric firearm injuries [10].

A surge in firearm purchases during the Covid-19 pandemic has resulted in approximately 30 million children now living in households with firearms [11]. The risk of suicide, unintentional injury and homicide, including among children and adolescents, is increased substantially by the presence of a firearm in the home [12–19]. Furthermore, the burden of firearm deaths is inequitably experienced by children, with disparities observed among gender, racial, geographic and socioeconomic groups, all of which have widened significantly in recent years [20]. The Centers for Disease Control and Prevention thus find that “Firearm injuries are a serious public health problem” [21] and in 2024 the US Surgeon General declared firearm violence in America to be a public health crisis [22].

The American Academy of Pediatrics (AAP) advises parents that the “most effective way to prevent unintentional gun injuries, suicide and homicide to children and adolescents, research shows, is the absence of guns from homes and communities”, explicitly advising “that the safest home for a child is one without guns” [23]. Recognizing that there may be circumstances where a child will be residing in a home with a firearm, the AAP states that: “If you decide to keep guns in the home, be aware that many studies show that teaching kids about gun safety, or to not touch a firearm if they find one, is not enough.” They then go on to provide advice about how to use and store the firearm to reduce the risk of harm to children:

All guns in your home should be locked and unloaded, with ammunition locked separately. Make sure children and teens can’t access the keys or combinations to lock boxes or gun safes. And remember not to keep loaded, unlocked guns in the car, or anywhere else on your property, either [23].

Consistent with this advice, a 2018 review of the literature on gun safety educational interventions found that there is limited evidence for their effectiveness, particularly in real-world settings, with the authors concluding that gun safety programs “do not improve the likelihood that children will not handle firearms in an unsupervised situation” [24]. Furthermore, parents often underestimate their child’s awareness of where a firearm is stored in the home, and many inaccurately believe that their child would avoid handling the firearm and instead alert an adult if they were to encounter one [25, 26]. Despite the importance of secure firearm storage (e.g., locked, unloaded, and separate from ammunition), a 2023 analysis of available data found that firearms used in unintentional injury deaths among children and adolescents were often stored unlocked and loaded and were frequently accessed from nightstands and other sleeping areas [27].

The most effective ways to protect children and young people from firearm injuries – strengthening of firearm control legislation, including restrictions on firearm design, marketing, availability and accessibility [28–31] – conflict with the interests of the firearm industry and its affiliated pro-firearm lobbying organisations such as the National Rifle Association (NRA), which seek to normalize and promote civilian firearm ownership [32, 33]. Furthermore, the issue of gun control is highly politicized and divisive in the US where there is an embedded ideological commitment to gun ownership, in part driven by the activities of the firearm industry which has sought to align the constitutionally enshrined right to bear arms with national identity, and ideas of masculinity and personal safety [32–34]. Historically, understanding of the causes of gun violence has been undermined by the 1996 Dickey Amendment (for which the NRA lobbied) prohibiting the use of federal funds to advocate or promote gun control [35]. It was not until 2018 that Congress issued a report explicitly stating that the Dicky Amendment does not prohibit the use of federal funds to support research on the causes of gun violence [35]. Within this context, the firearm industry, and associated groups like the NRA, represent an important commercial determinant of injuries, violence and inequities in the US and their practices need to be further scrutinized to prevent harm [5, 36].

There is a growing body of literature examining how the practices and products of commercial actors shape health, [37] and the corporate political activities adopted by health-harming industries (HHIs), such as tobacco, alcohol, and gambling industries, to influence regulatory environments, including strategies to “frame” perceptions of their products and set the terms of policy debates [38]. Framing strategies enable commercial actors to circumscribe what is perceived to be possible and preventable and influence what are accepted as legitimate policy responses and the values underpinning these, including in the context of the prevention of injuries and violence and public safety policymaking [38, 39].

The promotion and funding of education, particularly youth education programs, is a common element of HHIs’ corporate political acitvities [38, 40–42]. This practice enables commercial actors to disseminate an industry-favorable framing of a problem and associated solutions, to present themselves as benevolent public health experts, and to substitute for other more effective policy interventions that threaten their business interests (such as product or marketing bans) [38, 43]. Given the significant contribution of their products to avoidable mortality and morbidity, and their attempts to shape the relevant policy debates, the US firearm industry should be recognised as an important commercial determinant of health (CDOH) [5]. However, to date, the firearm industry has received comparatively limited attention from CDOH scholars [4, 5, 36]. The research we do have demonstrates that the firearm industry adopts similar framing strategies to those of other HHIs to promote the idea of personal responsibility, undermine evidence on the risks of widespread firearm accessibility and the effectiveness of firearm policies, and to conflate firearm restrictions with a loss of freedom, identity, personal safety and even American democracy [36].

The promotion of youth education and safety campaigns by the firearm industry, and industry-funded groups, has not been subject to detailed empirical analysis from a CDOH perspective, as has been the case for the tobacco, alcohol and gambling industries [40, 41, 43, 44]. This gap is notable given that industry-funded organisations like the NRA claim that this is an effective way of keeping children and young people safe from firearm injuries [45]. The NRA's flagship Eddie Eagle GunSafe^®^ education program was initially launched in 1988 and revised in 2015 [46, 47]. According to the NRA, the film-based program aims to educate children on how to respond if they find a firearm and “26,000 schoolteachers and law enforcement officers have taught the Program to over 32 million children” [47]. Despite a lack of independent evidence of its effectiveness in keeping children safe [24], the urgent need for decisive action to address firearm injuries among children in the US, and the conflicts of interest that emerge from the delivery of an industry-funded child safety program, there has been little scholarly attention paid to the NRA's strategies to promote and legitimize the ongoing delivery of the Eddie Eagle GunSafe^®^ program and the consequences of this.

The present study aims to fill the gap in the existing literatures on the firearm industry as a CDOH and on the content of industry-funded youth education programs. To this end, we analyze how, through the design, promotion and delivery of the program, the NRA frame (1) gun ownership, safety and the role of guns in society as part of their efforts to normalize firearm ownership, (2) the role of the Eddie Eagle GunSafe^®^ program as a firearm safety intervention; and (3) the role of the firearm industry and other actors within wider firearms policy debates. In so doing, we examine whether and, if so, how the program serves the corporate political interests of the firearm industry.

## Methods

This section describes the data collection process, the chosen analytical framework and how this was applied to the data.

### Data collection

Data were collected from two main sources: Eddie Eagle program-specific websites hosted by the NRA and purposively selected outputs from the NRA and its sub-divisions. The NRA hosts two websites dedicated to the Eddie Eagle GunSafe^®^ program: one intended for professionals (such as teachers and law enforcement officers) and parents [48], and one for children called the *Eddie Eagle Tree House* [49]. The textual content of these websites was downloaded in PDF form for the purposes of the analysis. Each webpage was accessed by navigating from the website’s home page using the tab functions and live hyperlinks. Where freely available, Eddie Eagle GunSafe^®^ program materials and newsletters were downloaded, and film-based materials and testimonials were viewed through the websites.

The NRA and its various sub-divisions produce several magazine or news outputs, two of which were purposively selected for identification of additional data relating to the Eddie Eagle GunSafe^®^ program. The news section of the NRA publication *America 1st Freedom*, described as an “official journal of the NRA”, was searched for additional data [50]. The term “Eddie Eagle” was applied using the search function producing a total of 78 articles of which 23, dating back to 2015, were identified as being predominantly focused on the program (i.e. the entire article, or a substantial proportion of the article, was dedicated to discussing some aspects of the program and its delivery, and/or the role of child firearm safety education). The same strategy was adopted to identify relevant articles produced by the lobbying arm of the NRA, the Institute for Legislative Action (NRA-ILA) [51]. This produced an additional 105 articles of which 50, dating back to 2001, were identified as being related to the program. This approach was not designed to be an exhaustive search for *all* articles produced by the NRA that discuss the program or gun safety education but was intended to assist the collation of a purposively selected sample of articles that would contribute to the examination of the types of public statements and claims made by the NRA about the program and its role in protecting children from firearm injuries beyond the program materials and dedicated websites. This approach enabled a more comprehensive analysis of the practices adopted by the NRA to promote the program and frame the role of gun safety education as a means of keeping children safe, while keeping the dataset manageable. All data were collected during the period November 2023 to February 2024.

### Analysis

A critical interpretive analysis was conducted supported by the application of published taxonomies of corporate political activities developed by Ulucanlar et al. [38]. These taxonomies catalogue practices that are adopted by HHIs to “secure preferential treatment and/or prevent, shape, circumvent or undermine public policies in ways that further corporate interests” [38]. The taxonomy of *framing* strategies and associated claims captures the ways commercial actors seek to construct public discourses which portray corporations as legitimate, authoritative and trustworthy actors whose framing of the problem and required solution – namely a minority of individuals whose so-called misuse of commercial products can be corrected through the adoption of industry-favourable individual-level interventions – are to be seen as the most logical and efficacious way of understanding and acting upon what they characterise as a trivial issue. Other policy actors who present different framings unfavorable to corporate interests are portrayed as illegitimate and biased participants in policy debates, who promote what is to be perceived as an unacceptable and unfounded explanation of the problem and required response [38].

The taxonomy of *action* strategies and associated mechanisms captures the practices that bolster these framing strategies, namely: accessing and influencing policymaking; using legal tools to obstruct policies; manufacturing doubt by shaping of the evidence base; mobilizing public support for corporate positions; displacing and substituting for public health; and reputation management to both bolster corporate images while discrediting public health actors [38]. Corporate actors modify their framing and action strategies, which are intimately linked and function synergistically, to adapt to dynamic social and political contexts, helping to reproduce or transform policy regimes in ways favourable to the corporation or an industry as a whole. This “hyper-adaptability” often manifests as contradictions in corporate arguments or actions [38]. For example, commercial actors may undermine public health framings by questioning the relevance of internationally obtained evidence while at other times drawing on their own data obtained from another context to legitimize their arguments and influence the policymaking process [38].

These taxonomies were used to examine the practices adopted by the NRA to promote and legitimize gun ownership and the Eddie Eagle GunSafe^®^ program and how these contribute to the corporate political interests of the firearm industry and its proponents. In the initial phase of the analysis, the first author read or viewed the collated documents and films, respectively, to gain familiarity with the content and identify dominant themes and practices. A closer second and third reading and viewing were then conducted to code the data guided by the taxonomies of framing and action strategies which were adapted as novel findings were identified. Specifically, the data were deconstructed, and textual or visual elements were assigned to one or more category of framing or action strategy described by Ulucanlar et al. [38]. This stage of the study was supported using an Excel spreadsheet to collate the coded data. At this stage, the coded data were reviewed by the second author to reach a consensus on the assignment of textual or visual elements to a given framing and/or action strategy category. Any disagreements in coding of the data were resolved through open discussion [52, 53].

Consistent with previous studies of industry-funded information and education programs, the coded data were then analyzed using a commercial determinants of health lens and drawing on framing and discourse theories to provide a critical explanatory account [54] of the functions played by, and interplay between, the identified framing and action strategies and their alignment with the political agenda of the firearm industry [40, 41, 44, 55, 56]. Contradictions in the NRA's overall strategy were noted as were departures from well-established standards of evidence and conceptualizations of child safety [40, 44]. In this way, the study applied a broader lens to the functions played by the delivery of an industry-funded youth education program, seeing its delivery not as a single practice, but constituting instead a network of interconnected, synergistic framing and action strategies.

## Results

**Fig. 1:**
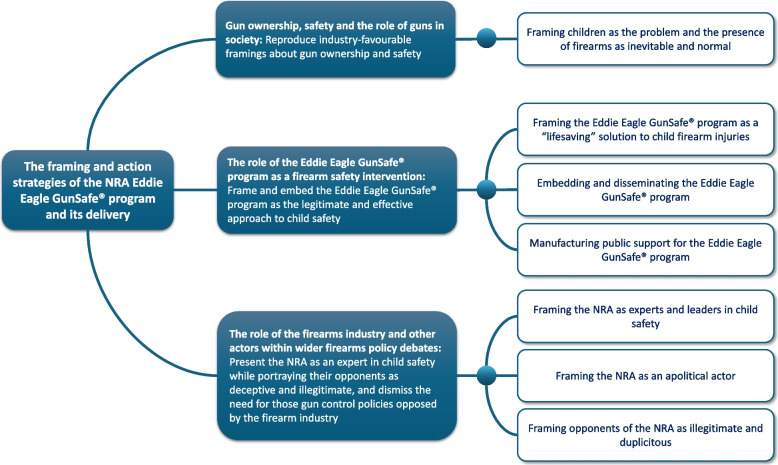
Schematic overview of their inter-related functions

The analysis identified how the NRA uses a number of inter-related and synergistic strategies to frame children’s behavioral responses to firearms as the problem (as opposed the widespread accessibility, availability and marketing of firearms and absence of mandatory safety measures) and the Eddie Eagle GunSafe^®^ program as the most logical, effective, and legitimate solution to keeping children safe from firearm injuries. Overall, the Eddie Eagle GunSafe^®^ program, and the practices used by the NRA to promote its delivery, serve to reproduce industry-favourable framings about gun ownership and safety, to present the NRA as an expert in child safety while portraying their opponents as deceptive and illegitimate, and to dismiss the need for those gun control policies opposed by the firearm industry. Each of these strategies and their functions are discussed further in the following sections and Fig. 1 provides a schematic overview of their inter-related functions.

### Gun ownership, safety and the role of guns in society

#### Framing children as the problem and the presence of firearms as inevitable and normal

The NRA asserts that is it not the widespread ownership and presence of firearms that should be understood as the problem but children’s behavioral responses should they encounter a firearm unsupervised. This framing of the problem re-enforces the idea that gun ownership is inevitable and desirable, and that it is both normal and unavoidable that children will encounter a firearm in their homes, playgrounds, mother’s handbags and parents’ bedrooms as depicted in the program’s materials (see next section); they just need to know what to do if this happens. The NRA thus rhetorically redefines the problem as arising from children’s behavior around guns, the presence and promotion of which are seen as unproblematic. The NRA also redefines the very concept of safety in a corporate friendly way. From public health and harm prevention perspectives, safety is understood as the creation of safe environments or products through the removal of hazards, and the adoption of safe designs and processes so that harm is unlikely to occur [57]. However, the NRA frames safety in a much more limited way: as something that is achieved by individuals responding to danger in the appropriate way. In this case, it requires children “knowing” what to do and acting upon that knowledge, against their natural childhood instincts and tendencies of curiosity, imitation, and risk-taking, to ensure their own safety. This is most evident in the way the support materials, provided as part of the program, instruct teachers to educate pre-K (also known as pre-Kindergarten or preschool, ages 3–4 years) to 4th grade (ages 9 to 10 years) children about the concept of safety (Table 1) [58–60].

**Table 1:** Lesson instructions on how to teach children about the concept of safety (source pre-K/Kindergarten, 1st/2nd grade and 3rd/4th grade Parent/Instructor Guides) [58–60]

STEP ONE: SAFETY DISCUSSION (10 MINUTES)
Begin by having a broad discussion about safety topics. The goal is to get children to understand the concept of safety and begin thinking about what defines safe and unsafe situations. Explain to students that safety is taking actions to avoid getting hurt and finding a safe place free from harm or danger.
Discussion Questions:
• What do you think safety is?
• Who are some people that help keep you safe?
• What decisions have you made to be safe?
• What decisions could you make to be safe?

The problem is further potentially downplayed and trivialized by means of omission, an important element of framing [61]: in the program materials analyzed there is no acknowledgement that, in the US, firearm injuries are a leading cause of death among children and adolescents, and that it is the presence of firearms in homes and communities that places children at risk of harm [12, 23]. Unlike the materials produced by the AAP, parents, teachers and other professionals are not informed that the most effective way to keep children safe is to have homes and communities free of firearms. Additionally, and consistent with efforts by other industries, such as the automobile industry, to deflect from the avoidably harmful nature of their products and policies governing their sale and use [62], trivializing language like “accident” is predominantly employed in the texts while terms like “violence” and “injuries,” which are adopted by the CDC and AAP, are used rarely.

### The role of the Eddie Eagle GunSafe^®^ program as a firearm safety intervention

#### Framing the Eddie Eagle GunSafe^®^ program as a “lifesaving” solution to child firearm injuries

By establishing children’s behavioral responses to firearms as the problem, the NRA is able to frame the Eddie Eagle GunSafe^®^ program as the preferred approach to child safety. The NRA describes the program as “a gun accident prevention program that for over 30 years has helped keep kids safe” [45]. The program’s main element is an eight-minute animation film which is accompanied by additional materials and activities (Table 2). According to the NRA, the program was designed in collaboration with relevant experts to keep children safe by teaching them what to do if they find a firearm:

The program was developed by a task force made up of educators, school administrators, curriculum specialists, urban housing safety officials, clinical psychologists, law enforcement officials and National Rifle Association firearm safety experts. It began in 1988 with one mission: teach children four simple, easy to remember steps so they know what to do if they ever come across a gun [45].

**Table 2:** Summary of freely available resources provided by each of the Eddie Eagle GunSafe^®^ program websites*

Website	Resources provided	Intended audience	Description
Professional/parent facing website https://eddieeagle.nra.org/	Information, teaching materials	Teachers and other professionals, parents	1. Information about the program and The Wing Team characters 2. Parents and FAQs sections 3. Parent and professional testimonials 4. Program-specific newsletters 2016–2023 5. The Eddie Eagle Film (main element of the program) 6. Two additional Sing-Alongs (Wing Team Sing-Along and Stop, Don't Touch Sing-Along) 7. Program resources: a. Pre K / Kindergarten Instructor Guide b. Pre K / Kindergarten Kids Activity Booklet c. 1st / 2nd Grade instructor guide d. 1st / 2nd Kids Activity Booklet e. 3rd / 4th Grade Instructor Guide f. 3rd / 4th Kids Activity Booklet g. Eddie Eagle Certificate 8. Information about grant funding and the Eddie Eagle mascot costume
Child facing website *The Eddie Eagle Tree House* https://www.eddieeagle.com	Child-facing films and activities	Children	1. The Eddie Eagle Film (main element of the program) 2. Lessons 3. Interactive introduction to The Wing Team 4. Other short *Storybook* films: a. Cooking up mischief b. Hide & seek c. Family secretes d. Skyathalon e. Lake trip f. Surprise 5. Sing-alongs 6. Colouring activities 7. An online trivia challenge 8. Workbooks

*Note: In addition to the materials examined here the program website has the following materials available for a fee, which were not included in the present study: Animated DVD, Student reward stickers, and a Parent's Guide to Gun Safety

The main film introduces Eddie Eagle, an animated American bald eagle (a symbol of American patriotism and identity), and his “Wing Team,” Fiona Falcon, Howie Hummingbird, Gary Goose, and Maya Guacamaya, who are intended to represent children of different personalities, ethnicities and genders [63]. In the film, Eddie Eagle and his Wing Team unexpectedly find a firearm in the playground where they are playing basketball [64]. The characters emulate what a child in the same situation should do by breaking into song including the program’s main message: “Stop! Don’t touch. Run away. Tell a grown-up”. The group do not touch the firearm, instead leaving the area to alert Eddie’s father and Fiona’s mother. The latter, Fiona explicitly states, will know what to do because she owns a gun and, the program materials tell us, carries this in her handbag [64]. The NRA states that:

Pre-k through fourth grade children [ages 3 to 10 years] will find this video engaging with its catchy songs, dance moves and entertaining dialogue-but most importantly, they’ll know what to do if they ever come across a gun [65].

The NRA frames this approach to firearm safety as being no different to other safety issues that children are taught about:

You talk about stranger danger, internet safety, fire drills and more with children...so why not include gun safety? The program makes no value judgments about firearms, no firearms are ever used, and it covers an important topic that needs to be addressed with kids. Like swimming pools, electrical outlets and lighters, firearms are simply treated as a part of everyday life. With firearms found in about half of all American households, it’s a stance that makes sense [45].

By aligning the issue of keeping children safe from firearm injuries with other issues of safety, the NRA's framing obscures the profound differences between a society choosing to have access to swimming pools and electricity, and the widespread marketing, availability and accessibility of firearms to civilian populations. It also overlooks the suite of other safety policies and measures that are taken to protect children from being harmed by these entities beyond just education, many of which are based on prevention and the removal or modification of hazards so that children are not placed at risk of harm. According to the NRA, the obvious response to the fact that firearms are “part of everyday life” [45] for children, and can be found in approximately half of all households in America, is not to reduce firearm prevalence and thus children’s exposure to the associated dangers, but to place the responsibility on children to manage the threat this poses to their safety. This framing is also highly misleading as it implies that the presence of firearms in American communities is natural, necessary and unavoidable, obscuring the fact that this is not the case in virtually every other high-income country in the world, with associated differences in firearm-related harms, and is the result of conscious political decisions. Given the assumptions made about the positions of guns in American society, the contention that the program does not make “value judgements” [45] about gun ownership appears highly contestable. Furthermore, the reassurance that “no firearms are ever used” [45] is questionable. While it is true that no firearms are discharged, they are still “used” in the sense that they are owned and carried by characters who feature in the program’s materials [49, 64].

The NRA frames their program as “lifesaving” and, at times, attributes downward trends in child firearm injuries in the US to the delivery of its program:

The effectiveness of the program is confirmed by declining gun accidents among children, its popularity with the schoolteachers and law enforcement officers who teach it, and testimonials that relate incidents in which children encountered guns, but because of what they learned in the Eddie Eagle program, they sought help from an adult and avoided potential injuries […] Firearm-related accidents among young children have been on a decline since NRA launched the Eddie Eagle program. It’s a testament to NRA's commitment to child safety and Eddie’s lifesaving message [66].

The NRA has also made statements about the program’s purportedly beneficial impact on children’s knowledge and safety throughout the period from which the data analyzed here is taken (2000 to 2023). Table 3 presents example quotes demonstrating how these statements have been employed over time and through different outlets of the NRA. Framing the program as “lifesaving” relies on an industry-favourable understanding of what counts as evidence of effectiveness. According to the NRA these include the organization “feeling” that the program has had a significant impact as well as anecdotal evidence about the popularity of the program with teachers and officials, or children reciting or acting out the program’s core message. For example, under the FAQs section of the program’s adult-facing website, the NRA states that:

the effectiveness of the Program is evident in several ways. First, fatal firearms accidents in the Eddie Eagle age group have been reduced by more than 80 percent since the program’s nationwide launch, according to the National Center for Health Statistics. NRA feels that gun accident prevention programs such as Eddie Eagle are a significant factor in that decline [47].

**Table 3:** Examples of statements made and/or disseminated by the NRA about the Eddie Eagle GunSafe^®^ program’s impacts

**Document** (by outlet and in chronological order from most recent to most distant when dated)	**Quote** (emphasis added)
Eddie Eagle GunSafe® Program adult facing website, home page	The Eddie Eagle Video Eddie Eagle and the Wing Team encounter a gun in a place that they didn't expect. Eddie helps his friends decide what to do to stay safe by reminding them of his favorite song. The Wing Team makes the right choice, but they still have some questions about gun safety. So they look to adults they trust for answers. Pre-k through fourth grade children will find this video engaging with its catchy songs, dance moves and entertaining dialogue-but most importantly, they'll *know what to do* if they ever come across a gun
Eddie Eagle GunSafe® Program adult facing website, “About” webpage	The Eddie Eagle GunSafe program is a gun accident prevention program that for over 30 years has *helped keep kids safe*
Eddie Eagle GunSafe® Program adult facing website, “For the Parents” webpage	Parents who accept the responsibility to learn, practice and teach gun safety rules will *ensure their child's safety* to a much greater extent than those who do not. Parental responsibility does not end, however, when the child leaves the home. That is why it is critical for your child to know what to do if he or she encounters a firearm. The Eddie Eagle GunSafe program has no agenda other than accident prevention – *ensuring that children stay safe should they encounter a gun*
Eddie Eagle GunSafe® Program adult facing website, “FAQs” webpage	Let your school administrators know that the Program is available and free. The Eddie Eagle GunSafe program is a worthwhile use of educators’ time whether you have five minutes or several days to cover the material. It’s about safety, and it’s important – because it could *save a child’s life*
Eddie Eagle GunSafe® Program adult facing website, Stop, Don't Touch Sing-Along description	This mantra is the very cornerstone of the Eddie Eagle Program. If you see a gun: Stop! Don't touch. Run away. Tell a grown-up. With a catchy song and fun dance moves, *your child will be sure to remember it*
NRA Eddie Eagle GunSafe® Program, Newsletter, winter, 2023	Holidays and winter breaks are approaching. It’s time to plan for class parties, school events, church and community events, and so many opportunities for sharing the *lifesaving* Eddie Eagle message!
	The future looks bright for Eddie Eagle in Cumberland County! The Sheriff’s Office with Officer Aytes plans to continue to bring Eddie Eagle and his *lifesaving* message to as many students as possible. We look forward to seeing the adventures he continues to have!
NRA Eaddie Eagle GunSafe® Programme, Newsletter, fall, 2023	She partnered with Knox County Sheriff Department and together they purchased the Eddie Eagle mascot costume for use with the presentations. She started by speaking with a local school agency and presented the material to the Superintendent for approval. They then identified a pilot location to present the program. The *success* at that location led them into presenting at the rest of Knox County School District. It was so well received that they then approached another nearby district to seek approval from their schools. They will begin implementing Eddie Eagle to all eleven Laurel County elementary schools this school year. This brings the total number of schools to 18 elementary schools receiving this *life-saving* message
NRA Eaddie Eagle GunSafe® Programme, Newsletter, 2019	Created in 1988 by past NRA President Marion P. Hammer, in consultation with elementary school teachers, law enforcement officers, and child psychologists, the program provides Pre-K through third grade children with simple, *effective* rules to follow should they encounter a firearm in an unsupervised setting: “If you see a gun: STOP! Don’t Touch. Run Away. Tell a Grown-Up.”
	Offered for free to educators and with readily available instructor’s guides online, teachers have everything they need to educate their students on how to stay safe. Thanks to local educators, hundreds of thousands of children *learn how to stay safe* if they encounter a firearm
	The Eddie Eagle GunSafe Program is proud to announce that it has now reached over 32 million children with its *life saving* message! With over three decades of teaching safety, the Eddie Eagle program continues to move forward with its mission and is *proving* to be as *successful* as ever
	We look forward to working more with Ms. Robinson in the future. She has done so much to help spread Eddie Eagle’s *lifesaving* message of “Stop! Don’t touch. Run Away. Tell a Grown-up.”
	Thank you to Orelene Rivers and other educators like her for their compassion, dedication, and support which will help Eddie Eagle reach, educate, and *save* more children
NRA Eaddie Eagle GunSafe® Program, Newsletter, 2018	The Eddie Eagle GunSafe® Program is celebrating its 30th anniversary in 2018! In the program’s three decades of outreach, more than 30 million children across the United States have learned NRA simple yet *effective* firearm accident prevention principles
	Aside from law enforcement events, Mr. Carter attends other public events, such as NRA Weekend at Cabela’s and even to local children’s hospitals where he spreads Eddie Eagle’s message through videos, activity books, and other materials. Mr. Carter has shown exceptional dedication to *keeping the children of Texas safe by drastically increasing Eddie Eagle GunSafe® Program’s presence in the state*
	“The *effectiveness* of the program is confirmed by declining gun accidents among children, its popularity with the schoolteachers and law enforcement officers who teach it, and testimonials that relate incidents in which children encountered guns, but because of what they learned in the Eddie Eagle program, they sought help from an adult and avoided potential injuries,” said Eric Lipp, NRA National Community Outreach Manager. “*Firearm-related accidents among young children have been on a decline since NRA* *launched the Eddie Eagle program. It’s a testament to NRA* *commitment to child safety and Eddie’s lifesaving message*.”
	Students at Pontiac Elementary School in Elgin, SC are enjoying the Eddie Eagle GunSafe® Program. “STOP! Don’t Touch. Run Away. Tell a Grown-up.” is what the children are saying at the Pontiac Elementary School. Approximately 40% of U.S. households have firearms and Eddie Eagle’s message, “STOP! Don’t Touch. Run Away. Tell a Grown-up.” is *helping prevent* unintentional firearm fatalities with children
NRA Eddie Eagle GunSafe® Program, Newsletter 2017	According to the National Center for Health Statistics, accidental firearm-related deaths among children in Eddie Eagle’s targeted age group have declined more than 80% since the program’s launch. “The message is simple, easy to remember and fun for kids to learn,” said Lipp. Created in 1988 by past NRA President Marion P. Hammer, in consultation with elementary school teachers, law enforcement officers, and child psychologists, the program provides pre-K through fourth grade children with simple, *effective* rules to follow should they encounter a firearm in an unsupervised setting: “If you see a gun: STOP! Don’t Touch. Run Away. Tell a Grown-Up.”
	Eddie Eagle staff spoke with school representatives in-depth about the mission of the program and the variety of ways educators could implement it into their schools. From reading storybooks to taking a safety quiz, Eddie and his Wing Team deliver an important message that *all kids can benefit from*: STOP! Don’t Touch. Run Away. Tell A Grown-up
	Sgt. Blue and her team are continuing to share the Eddie Eagle message whenever they get the chance. She hopes to bring materials to area schools and stresses that “this is one of the busiest times of the year for kids to wander outside or inside the home,” and they will be doing all they can to prevent accidents from happening. She also participated in her community’s National Night Out in August. Sgt. Blue and the Eddie Eagle Program “encourage everyone to sign up, get the materials, and *save a life before it is too late*.”
NRA Eddie Eagle GunSafe® Program, Newsletter, 2016	Since introducing the program in 2009, Ms. Blount has reached over 4,000 children with Eddie’s *lifesaving* message. Following the lesson, it is not uncommon to hear the students singing the chant and doing the motions while they are on the playground or in the hallway
	“It’s about safety—nothing more, nothing less,” said Wayne LaPierre, NRA Executive Vice President. “And when it comes to our children, nothing is more important.” The NRA is the leader in teaching firearm safety and the new Eddie Eagle will *teach millions of children how to potentially avoid an accident*
*Did the Mainstream Media Just Endorse an NRA* *Program*, America’s 1st Freedom news article, August 05, 2023	The NRA has been doing yeoman’s work in teaching gun safety to children for decades and has known this “trick” all along. Since 1988, the NRA Eddie Eagle GunSafe Program has taught more than 32million children that if they see a gun, “Stop! Don’t Touch. Run Away. Tell A Grown-up.” In the process, the program has *undoubtedly saved many lives*
*Virginia County School Board Moves to Educate Youth on Safe Gun Handling*, America’s 1st Freedom news article, March 12, 2020	“The Eddie Eagle program has been helping schools teach firearm accident prevention for over 30 years, and we are thrilled to possibly be able to help the children of Culpepper County learn Eddie’s safety message *and help keep them safe*,” said Eric Lipp, the NRA's community outreach national manager. “The Eddie Eagle program has one mission, and that’s helping children stay safe, which we hope all elementary schools can support.”
*Eddie Eagle Soars Online*, America’s 1st Freedom news article, February 16, 2020	In its early days, an adult would don an Eddie Eagle mascot costume and convey the GunSafe message, with Friends of NRA monies funding grants for the costumes and supporting materials. Today, Eddie’s message is more easily available, with free downloads of materials at the Eddie Eagle Treehouse, an interactive website that offers a new animated video, sing-along songs and more that gets the message across in a way that *keeps the attention of children* in the target age group of 5–9 years old. The relatively new online resources should boost Eddie Eagle’s reach beyond the million-plus annual reach the program has averaged in its first 32 years
	The effectiveness of Eddie’s message is measurable. The Centers for Disease Control and Prevention have reported that the number of unintentional firearm fatalities among children has declined about 80% since the program’s inception, and gun-safety programs are *undoubtedly a major component in that drop*
	Eddie also has garnered a solid following of supporters among educational and law-enforcement professionals. Endorsements by groups such as the National Sheriffs’ Association, the American Legion, the Police Athletic League, the National Association of School Safety and Law Enforcement Officers, and the American Association of American Educators support the wide-ranging appeal of the program. Throughout its existence, countless testimonials *confirming that children have applied the lessons in real-life situations, thus avoiding injury, have also come in*
*Eddie Eagle Has Taught 31 Million Children Gun Safety*, America’s 1st Freedom news article, November 12, 2018	“Since our founding, the NRA has been committed to firearm safety, responsibility and education,” said NRA Executive Vice President and Chief Executive Officer Wayne LaPierre. “Those important concepts are the hallmarks of the Eddie Eagle GunSafe Program. Eddie’s *incredible success is proof that proactive accident prevention education works, and works well.* Our children are our future, and it’s our responsibility to teach them how to stay safe. To that end, the NRA will continue to work with community leaders to reach youths across our great nation.” The method has been well-received across the nation, with governors and/or legislative bodies from about half the states proclaiming its merits and recommending the program
*Atlanta Newspaper Op-Ed: NRA* *Helps Keep Kids Safe*, America’s 1st Freedom news article, April 28, 2016	Given the yearly ramping-up of anti-gun rhetoric in the media during the week leading up to the NRA Annual Meetings and Exhibits, it was refreshing to see an op-ed in the Atlanta Journal-Constitution Yesterday acknowledging the work NRA does to promote safety. Penned by Leslie Deets—owner of Sharp Shooters USA, an indoor shooting range, retail store and training facility—the article, titled “ NRA Work Helps Keep Kids Safe Around Guns,” cites CDC data for the last two decades showing that even as gun ownership has risen, gun fatalities among young children have dropped 65 percent. “*I credit one program in particular for the record-low number of accidental shootings among children: the National Rifle Association’s Eddie Eagle GunSafe Program*,” Deets said. In the last 30 years, the program—which is nonpartisan and makes no value judgment about firearms—has taught nearly 30 million children what to do if they find a gun. “The message is direct, simple and easy for children to remember. *I am convinced these four easy steps have saved countless young lives*,” Deets said
*Justice Department Funds Grant For Child Gun Safety Education*, America’s 1st Freedom news article, September 17, 2015	What’s more, NRA's Eddie Eagle GunSafe® program has been in operation more than a decade longer than NSSF’s Project Child Safe and reached more than 28 million kids with its comprehensive curriculum through schools nationwide. Kids love it, parents praise it, and *it’s helped reduce accidental firearm fatalities to the lowest levels ever recorded*. So where’s the NRA's federal grant?
*Kansas: House to Vote on Firearm Safety Education Bill*, NRA-ILA news article, March 02, 2023	NRA's Eddie Eagle GunSafe Program has been teaching firearm-accident prevention for over 30 years, *to help keep our children safe.* As a result, the program has reached over 32 million children in all 50 states, plus Canada and Puerto Rico
*NRA Celebrates Halloween by Helping to Teach Firearm Safety in America*, NRA-ILA news article, October 28, 2015	“Halloween offers a great opportunity to talk about safety with our children, and the NRA is thrilled that so many communities are using the chance to teach firearm safety,” said Amy Hunter, spokeswoman for the National Rifle Association. “The ‘Trick or Treat with Eddie Eagle and the Wing Team’ promotion is fun, informative, and *it saves lives*.”
	Firearms are found in approximately 40 percent of all American households. Even if there are no guns in your own home, there may be guns in the homes of friends that your children visit. To *ensure their safety*, your children must be trained what to do if they encounter a firearm. The Eddie Eagle program has no agenda other than accident prevention – *ensuring that children stay safe should they encounter a gun*. The program never mentions the NRA. Nor does it encourage children to buy guns or to become NRA members
	According to the Centers for Disease Control and Prevention, unintentional firearm fatalities among children of the Eddie Eagle program’s targeted age group have declined approximately 65 percent in the last twenty years. Firearm accident prevention programs such as Eddie Eagle are a *significant factor in that decline*
*New Jersey: Cape May County Sheriff introduces Eddie Eagle program*, NRA-ILA news article, November 05, 2009	Cape May County Sheriff Gary Schaffer is pleased to announce that the Sheriff's Office is offering a new educational program on gun safety for children from pre K to grade 3. Sheriff Schaffer said, "The purpose of the program isn't to teach whether guns are good or bad, but *rather to promote the protection and safety of children*."
	Indeed, Eddie Eagle has never been about politics or promoting any particular view of guns. NRA does not derive any revenues from the program. It’s simply *one way to help prevent accidents and protect kids*
*Gun safety programs are casualty of time crunch at area schools*, NRA-ILA news article, March 10, 2008	Outreach efforts such as the Eddie Eagle GunSafe Program—applauded by the National Safety Council and Association of American Educators, and endorsed in 1996 by the Idaho Legislature and Gov. Phil Batt—*helped lower child deaths due to unintentional firearm injuries from 247 in 1987 to 63 in 2004*, according to numbers from Safe Kids USA
*Eddie Eagle Teaches Students About Gun Safety*, NRA-ILA news article, May 03, 2006	Thanks to Officer Ed Dye and Eddie Eagle, primary students at three Pierce Township schools *know* what they should do if they ever find a gun
*NRA Victories: Eighteen Million Safer Kids*, NRA-ILA news article, July 27, 2006	When Paul and Kathryn Walters of Gladstone, Mich., moved into a new house with their three children, they didn’t know the previous owner had left a .22 bolt action rifle and a few rounds of ammunition in a closet corner where neither parent could fit. Seven-year-old Michelle Walters and twin four-year-old siblings Samantha and Christopher found the gun during a game of hide and seek. Michelle immediately commanded the younger children, “Stop! Don’t Touch. Leave The Area. Tell An Adult.” A Life-Saving Lesson The Dennet and Walters families—and countless others—*credit the NRA's* *Eddie Eagle GunSafe Program for teaching their kids how to prevent a potentially fatal accident*
	The NRA worked with schoolteachers and administrators, clinical psychologists, law enforcement officers, education specialists and firearm experts to develop a message *that’s readily understood by children* and easily taught by any adult: “If You See A Gun: stop! Don’t Touch. Leave The Area. Tell An Adult.”
	Among children in the Eddie Eagle age group, fatal firearm accidents have been reduced more than two-thirds since the inception of the program, according to the National Center for Health Statistics. What’s more, a 2001 study published in the Journal of Emergency Nursing Online named Eddie Eagle *the most effective among the more than 80 programs evaluated, drawing a distinct correlation between the Eddie Eagle Program and children’s lives saved*. Statistics aside, it’s the volume of testimonials that the NRA receives each year from parents that *proves* the true value of the Eddie Eagle Program
	In fact, when formally endorsing the Eddie Eagle GunSafe Program in March 2002, then NSA President Sheriff John Cary Bittick said, “We are proud to partner with the National Rifle Association on this very important issue, and we would like to express our full support for this program.” The Eddie Eagle GunSafe Program has also garnered praise from 49 state legislatures and/or governors, who have urged their respective state school systems to implement the *life-saving message the program offers*. And the Community Service Division of the National Safety Council recognized the tremendous contribution that the Eddie Eagle Program has made in *keeping kids safe* by awarding program creator Marion P. Hammer with one of its highest honors, the Community Safety Award Citation for Outstanding Community Service

The NRA frames long-term declines in firearm fatalities among children reported by the CDC as evidence of the effectiveness of the program’s message. According to the NRA, such declines are “undoubtedly” in part driven by the delivery of gun-safety programs:

The effectiveness of Eddie’s message is measurable. The Centers for Disease Control and Prevention have reported that the number of unintentional firearm fatalities among children has declined about 80% since the program’s inception, and gun-safety programs are undoubtedly a major component in that drop [67].

However, no evidence is supplied for such a causal claim, which relies instead on the intuitively appealing but logically fallacious form of “post hoc ergo propter hoc” reasoning. Claims of beneficial impact thus enable the NRA to frame the acceptable and legitimate standards of evidence and proof for establishing what is effective in keeping children safe. The NRA also stresses that beyond such supposed statistical evidence, parent testimonials provide proof of the program's benefit to child safety: "Statistics aside, it’s the volume of testimonials that the NRA receives each year from parents that proves the true value of the Eddie Eagle Program" [68].

#### Embedding and disseminating the Eddie Eagle GunSafe® program

The NRA acts to normalize and embed their program as the legitimate response to child firearm safety issues and promote its dissemination. These practices include influencing the policymaking process and manufacturing public support, as consistent with Ulucanlar et al.’s taxonomy of action strategies [38]. The first of these actions is evident in the way the NRA repeatedly attempts to influence the passage of State-level legislative bills related to gun safety education. These bills essentially stipulate for local school boards to issue standards for firearm safety curricula if a school decides to educate their students on this topic. Some of the bills specifically name the Eddie Eagle GunSafe^®^ program as the standard on which to base teaching younger school-aged children about firearm safety. Through the news sections of their *America 1st Freedom* publication and the NRA-ILA, the NRA provides regular updates on the passage of these bills through State legislators. For example, the NRA provided ongoing coverage of the passage of bill H.B. 791 through the legislative process in Maryland, with one article explicitly acknowledging the organization’s backing of the bill and stating that the bill’s adoption should be seen as a “victory” *for* the State’s citizens:

In a tremendous victory for the citizens of Maryland, today the House of Delegates passed a bill on second reading that will provide for gun-safety education in all schools. H.B. 791, sponsored by House Speaker Casper R. Taylor Jr. and backed by the National Rifle Association, gives power to the counties when it comes to deciding which gun-safety program to implement [69].

The same article went on to explain that, according to the NRA, the bill’s passage should also be seen as a “victory” *over* those who support alternative legislation unfavorable to the firearm industry:

Today marks a victory against organizations like Marylanders Against Handgun Abuse, who attempted to hijack this legislation in favor of anti-gun rights programs. H.B. 791 puts gun-safety where it should be, in the hands of the local school boards, not the state. Counties will be able to select the best program for their community and culture to ensure that children are safer than ever around firearms [69].

The coverage of these legislative processes thus facilitated the dissemination of NRA framings about who should be seen as legitimate, credible and trustworthy actors and the role of education programs, like the Eddie Eagle GunSafe^®^ program, as a means to “ensure” the safety of children from firearm injuries. Several reports encouraged the reader to act themselves by contacting their local representative to express their support for these bills, with some articles containing a “click here take action” button that facilitated written communication with State Representatives. At times, this form of legislation was explicitly referred to by the NRA as “pro-gun”:

Two bills sprinted through the legislative finish line yesterday as the Senate passed two pro-gun measures which were subsequently approved by the House. Omnibus legislation, House Bill 2058, and Eddie Eagle Gun Safety legislation, House Bill 2089, now head to the desk of Governor Laura Kelly for her signature [70].

Some articles created a sense of urgency if votes or opportunities to overturn a veto were imminent and condemned the actions of those who had voted against such bills. For example, over the period 2021 to 2023 the NRA repeatedly reported on the passage of several bills of this type through the Kansas legislative process all of which failed to be adopted. In the most recent iteration of the events the NRA used their reporting to frame this as a loss to promoting children’s safety and to portray the organization as a partner in prioritizing this agenda:

Unfortunately, this is the second time Governor Kelly has vetoed this important bill, despite its overwhelming bipartisan support in both chambers. Our children's safety should always be a top priority, and the Eddie Eagle GunSafe Program has been doing just that for over 30 years, reaching over 32 million children across the US, Canada, and Puerto Rico. **Please take action now and contact your lawmakers to encourage them to vote for the veto override. Use the link below to take action and make your voice heard. Then, share this alert with your family and friends, and ask them to do the same. Together, we can ensure that our children receive the education they need to stay safe around firearms. [...] Thank you for your support in this critical matter** (bolded text in original) [71].

#### Manufacturing public support for the Eddie Eagle GunSafe^®^ program

Beyond eliciting backing for specific forms of State legislation, the NRA acts to manufacture public support for their program by providing positive case studies of the program’s delivery across the US. For example, through the program’s website, the NRA provides a program-specific newsletter, *The Eagle Eye*, which features articles about educators, law enforcement officers and other actors who deliver the Eddie Eagle GunSafe^®^ program in their communities. Similar articles are repeatedly published through the news section of *America 1st Freedom* and NRA-ILA. The dissemination of these stories, some representing summaries of favourable media reports that provide links to the original source, gives the impression of widespread support for the program and benefits to children’s safety, and buttresses the framing of who is to be seen as a trustworthy actor; those who support the delivery of the program, including industry donors, are framed as collectively taking the logical and commendable approach to keeping children safe from firearms:

Volunteers for the Eddie Eagle program come from diverse backgrounds, but they share a commitment to keeping children safe. Those involved include NRA members, teachers, law enforcement officers, and community activists who teach the program, as well as private donors and Friends of NRA volunteers who raise funds to help pay for the program’s educational materials. More than 26,000 educators, law enforcement agencies, and civic organizations have taught the program since 1988 [72].

One story dedicated to recognizing a law enforcement Captain “for his extraordinary support and dedication in teaching children how to be safe around firearms, using the Eddie Eagle GunSafe^®^ Program” [73] explained that the program had been adopted by the local Sheriff’s Department in response to an incident in a school with the aim of preventing future incidents:

The Sheriff’s Department adopted the Eddie Eagle Program after an incident occurred at an elementary school about a year and a half ago. Since then, the department wanted to introduce the Eddie Eagle Program to other schools to prevent any future incidents. Luckily, the local principals and teachers have welcomed the opportunity to teach gun safety to children with open arms [73].

The program also recruits children to help build public support by encouraging teachers to deliver a letter writing activity. This activity requires children to write a reassuring letter explaining to their parents that they have watched a film about what to do if they find a gun and therefore “know” what to do to keep themselves “safe” in such a situation (Table 4a) [58, 60]. The instructor’s guide advises that:

If you do not choose to complete the group letter writing activity, send home the provided letter (also located in the back of this guide) and encourage students to continue the conversation about gun safety with their families [58, 60].

This second form of the letter (Table 4b) similarly disseminates the framing that exposure to the program’s main film component is effective at ensuring a child will “know” what to do if they find a gun and by implication will act on this knowledge to ensure their own safety [58–60].

**Table 4:**
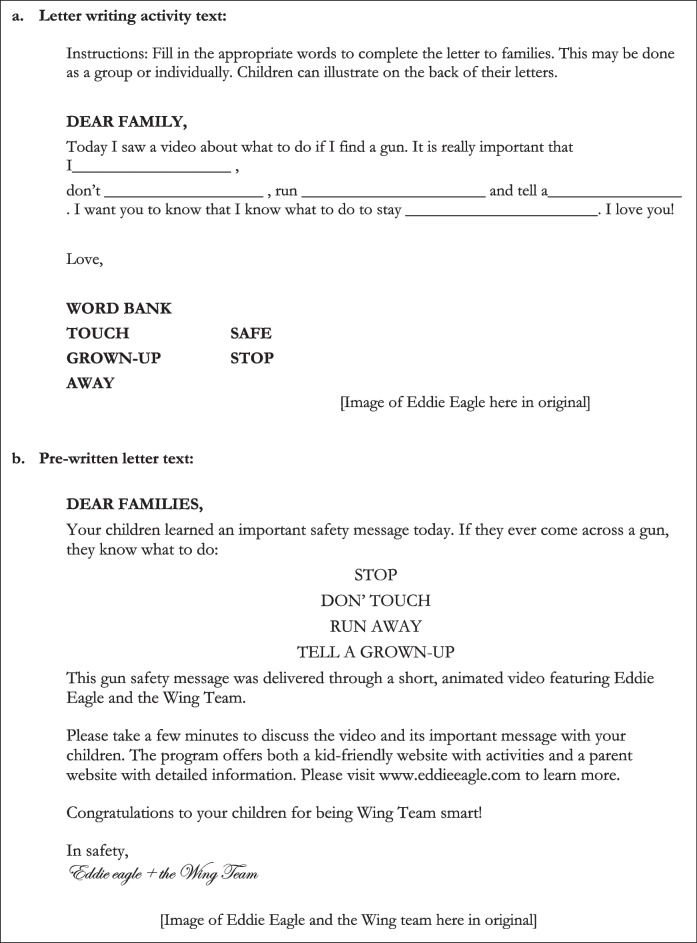
Letter writing activity (a) and letter to parents (b) provided as part of the Eddie Eagle program materials (Source Parent/Instructor Guide Pre-K and Kindergarten https://eddieeagle.nra.org/program-resources/program-materials/)

* Note the same letter to parents is included in the instructor guides for pre-k / Kindergarten, 1st/2nd grade and 3rd/ 4th grade but the letter writing activity differs in the latter

Dissemination of the program is further facilitated by the NRA who make the program’s film-based and supporting textual materials freely accessible via the two program-specific websites. Funding grants are also available to support the delivery of the program locally. The NRA promotes their grant giving scheme via the program’s website, newsletter, and *America 1st Freedom* news articles. The program website states that: “Schools, law enforcement, hospitals, day care centers and libraries are eligible for grant funding…Grants are generously made possible by Friends of NRA fundraising events” [47]. Friends of NRA is the fundraising arm of the NRA Foundation which receives funds from major firearm industry actors including, Ruger^®^, Smith & Wesson, O.F. Mossberg & Sons, Colt^®^, Sig Sauer, Springfield Armory USA and Savage Arms, among other companies [74]. Notably, many of these companies manufacture high capacity handguns, and in the case of Colt^®^, Sig Sauer, Springfield, Ruger^®^, Smith & Wesson, they also produce assault-style semi-automatic rifles. Handguns and assault-style semi-automatic rifles are common causes of firearm-related injury and often used in mass shootings [75]. By publishing accounts of the program’s delivery, as described above, the NRA disseminates the message that these grants are enabling the delivery of their “lifesaving” program:

In an effort to make sure children are kept as safe as possible from accidental shootings, Officer Donnelly applied for a grant sponsored by the National Rifle Association. Through the Eddie Eagle gun safety program, Donnelly's goal was to bring a message to about 450 children in kindergarten through third grades by Oct. 14 [76].

These action strategies enable the NRA to “displace and usurp public health”, a key element of the taxonomy developed by Ulucanlar et al [38]. By promoting their industry-funded education program as a widely supported “lifesaving” prevention intervention, the NRA can present a less effective, industry-favourable, individual-level, education-based measure as the common-sense approach to harm prevention, in place of more effective policy measures unfavorable to the industry. These actions function to reproduce the idea that widespread firearm ownership is normal, inevitable and uncontestable, and that children’s knowledge and behaviors should be the focus of firearm safety interventions. The NRA thus presents itself, and its “generous” industry backers, as acting to protect the safety of children and further burnish their image by association with respected and trusted individuals and institutions, like teachers, schools and law enforcement officers. The organization is then able to tarnish the reputation of those who question the delivery of the program or advocate for firearm policy reform by framing them as duplicitous, undermining of children’s safety, and politically motived, as detailed in the following sections.

### The role of the firearm industry and other actors within wider firearms policy debates

#### Framing the NRA as experts and leaders in child safety

The NRA's framing presents the organization as a disinterested actor which is to be understood as a source of expertise in firearm safety and whose actions help to protect children rather than representing the interests of the firearm industry:

Neither Eddie nor any members of his Wing Team are ever shown touching a firearm, and there is no promotion of firearm ownership or use. The NRA does not make any sort of profit off the program, nor does it intend to. The goal of the Eddie Eagle GunSafe^®^ program is to help prevent accidents and keep children safe [45].

In a 2018 article announcing that the program had reached 31 million children since its creation in 1988 the NRA Executive Vice President and Chief Executive Officer Wayne LaPierre was quoted as saying:

Since our founding, the NRA has been committed to firearm safety, responsibility and education […] Those important concepts are the hallmarks of the Eddie Eagle GunSafe Program. Eddie’s incredible success is proof that proactive accident prevention education works, and works well. Our children are our future, and it’s our responsibility to teach them how to stay safe. To that end, the NRA will continue to work with community leaders to reach youths across our great nation [77].

The article went on to state that “[t]he method has been well-received across the nation, with governors and/or legislative bodies from about half the states proclaiming its merits and recommending the program.” [77]. In another article reporting on an NRA initiative to promote the education program during Halloween, a NRA spokeswomen directly cited the Eddie Eagle program as evidence of their “world” leading efforts to promote firearm safety:

No other organization in the world does more to promote firearm safety than the National Rifle Association […] and the Eddie Eagle Program is a prime example of that [78].

In this way the NRA frames its activities as evidence that they work with trusted and credible actors and organisations to deliver an effective, education-based program that is protecting children and is widely embraced across the country.

However, analysis of the data reveals how the NRA's claim to expertise and impact is contradicted by their use of anecdotes as “proof” of their programs effectiveness in keeping children safe and the approach to the program’s delivery as a safety intervention. The main film is considerably longer than other films used in independently designed and evaluated programs, the program does not include an active behavior skills training component (an approach shown to be more effective than passive learning styles [24]), and the adult-facing materials advise instructors and parents to use as much or as little of the additional materials as they like and no specifications are provided as to how often a child must watch the main film or recite or act out its message in order to stay “safe” around firearms. In contrast, it is well established that safety interventions should be delivered and implemented in accordance with what is known to be effective and deviations from what has been shown to be effective in formal evaluations will affect the impacts of any invention and usually warrant further evaluation.

Most notably, the NRA launched a new version of the program in 2015 with two key changes being the introduction of the Wing Team characters (described above) and a revised core message: “Stop. Don’t Touch. Run Away. Tell A Grown-up.” To justify the change in messaging, the NRA explains that:

Eddie’s original mantra was “Stop! Don’t touch. Leave the area. Tell an adult.” However, during research, the NRA discovered that children had a hard time remembering words they didn’t use regularly—especially area and adult. The updated mantra integrates more kid-friendly words and phrases like run away and grown-up, and recall on the mantra has improved as a result [47].

The NRA also explains what underpins the design of the new film and the Wing Team:

The NRA held 22 focus groups in 12 cities across the country. These groups included superintendents, principals, teachers, parents and children from various ethnicities, genders and socioeconomic backgrounds. Groups reviewed a variety of storylines and characters, which were adapted based on feedback. The NRA is confident that the final product is approachable and relatable for all [47].

However, the NRA does not cite any evidence in support of these statements. Perhaps even more importantly it does not examine the implications of these findings for the claims they make about the program being effective since 1988, nor what these findings imply with regards to its likely inequitable impacts prior to these revisions. Instead, they simply frame these revisions under the reassuring label of “modernization”, and state that the changes are immaterial as their main aim of keeping children safe has not changed. The organization has proceeded to disseminate statements that conceal the substantial changes made to the program even in response to those who question the program’s legitimacy. For example, in an article written in response to a critique of the program by American television host and film producer, Steve Harvey, the NRA countered this by stating that the program had been delivering its life-saving message (citing the second version) since 1988, absent any reference to the revisions to the program and its tagline:

Steve Harvey used an episode of his daytime talk show […] to ridicule the NRA's longstanding gun safety program. He asked, “What’s a better way to keep kids from guns—laws that punish adults who let children get their hands on firearms, or is it Eddie Eagle?” The comedian then went on to mock the mascot and question an NRA claim that the Eddie Eagle GunSafe program has been credited with an 80-percent reduction in child firearm accident fatalities. Harvey should have done a bit more research […] since it began in 1988, the NRA's gun accident prevention program has been widely successful and adopted throughout the nation. To date, it has taught its life-saving message of what to do if children come across a gun— *Stop. Don’t Touch. Run Away. Tell A Grown-up*—to more than 28 million children in all 50 states, plus Canada and Puerto Rico. So who’s laughing now? [79].

The NRA's framings and the design of the program thus counter decades of public health evidence on the delivery of effective complex interventions and evidence on the drivers of firearm injuries among children. Such practices are clearly contradictory to the claims made by the NRA to be delivering an effective program simply because they claim to be committed to child safety.

#### Framing the NRA as an apolitical actor

The NRA presents itself as a neutral actor whose apolitical education program “neither offers nor asks for any value judgment concerning firearms” [47]. Indeed, the NRA explicitly states that “Eddie Eagle has never been about politics or promoting any particular view of guns. […] It’s simply one way to help prevent accidents and protect kids” [80]. As explained above, these assertions are contestable given the program's underpinning assumptions about firearm ownership. Yet the NRA frames child firearm safety as an apolitical topic that is no different to other safety issues and discredits those who question the NRA's activities by labelling them as “anti-gun” actors who subvert the topic of child safety for political gain:

Anti-gun groups continue to be harsh critics of the program, apparently because it’s preferred over their anti-gun “safety” curricula. But the Eddie Eagle GunSafe Program makes no judgment call as to whether guns are good or bad. And Eddie himself never touches a firearm [81].

In the same article the NRA provides what they describe as an example of such “anti-gun” efforts:

In a recent example, an organi-zation called pax attempted to block efforts by District Attorney Edward Jagels to introduce Eddie Eagle to all kindergarten students in Kern County, Calif. pax attacked the program in a letter to the school board and anti-gun zealot Dr. Arthur Kellerman criticized it on fox News. But Jagels and School Superintendent Larry Reider countered the criticism and put the safety of children over political gain. Today, all the district’s principals have received Eddie Eagle program materials for use in their schools [81].

However, the NRA also stated that their Eddie Eagle GunSafe^®^ program was created as a counter to what they described as the “flooding” of schools with “anti-gun propaganda” under the guise of a safety curriculum:

The Eddie Eagle GunSafe Program was the brainchild of NRA past-President Marion P. Hammer in response to anti-gun propaganda disguised as “safety” curriculum flooding our nation’s elementary schools [68].

#### Framing opponents of the NRA as illegitimate and duplicitous

The NRA frames those who question their activities and the role of gun safety education as “anti-gun” or even “dangerous”. For example, an article published by the NRA in response to the publication of a report on the evidence underpinning child firearm safety education explained that:

A recent “study” put together by a collection of anti-gun “researchers,” and funded by a foundation with a long anti-gun history, has come to the shocking and dangerous conclusion that programs that teach children how to be safe when firearms are present are ineffective and should be abandoned. The **David and Lucille Packard Foundation** has funded several biased, anti-gun studies in the past, and provided significant financial support to the anti-gun extremist organization, the "**Million Mom March**" (MMM) […] The list of anti-gun "researchers" included in the "study," coupled with the source of the funding, should be enough to tell most clear-thinking individuals that this latest effort was intended simply as another vehicle to promote the same anti-gun policies that have been championed for many years by the gun-ban movement (bolded text in original) [82].

The article went on to state that “[i]t is unlikely the Packard Foundation’s position opposing gun-safety programs will be supported by anyone but the truly anti-gun” [82]. In this way, the NRA frames those who oppose youth education as a means to keep children safe from firearms as holding biased views on gun policy and are, therefore, to be characterized as untrustworthy actors. This also enables the NRA to frame measures they oppose as the agenda of such “anti-gun” extremists, and the abandonment of firearm safety education as placing children’s lives at risk:

Perhaps the most dangerous and disturbing conclusion of the Foundation is the ridiculous notion that firearm safety programs have “limited effectiveness.” Should any policy makers take this outrageous position to heart, and should this lead to the abandonment of even one firearm safety program, it could quite easily lead to the tragic loss of life because a child was not exposed to an important safety message [82].

The organization focuses particularly on undermining the credibility of individuals and advocacy groups led by mothers who raise concerns about the program’s delivery and/or who advocate for strengthening of firearm laws. For example, the NRA publishes articles that frame mothers who question the delivery of the program as emotional and phobic individuals who do not represent mainstream views:

Showing that the depths of some individuals' hoplophobia know no bounds, CBS11TV.com (Dallas-Fort Worth) recently ran a story on a mother whose knee-jerk reaction against firearms was so overwhelming that even *pictures of firearms* in NRA's Eddie Eagle GunSafe Program's coloring book, sent her into a tizzy…The Garland, Tex. mom brought home the coloring book, apparently unaware of its content. When she learned what the educational book was, she condemned not only it, but NRA as well: “Not only do I think it's inappropriate to provide this information to my five-year-old, but this is a program published by the NRA.” Published by NRA—heaven forbid! […] Luckily, better commonsense prevailed at the elementary school […] School officials point out that the safety curriculum is 10-years-old, and this was the first complaint the district has received since it began. Let's hope it's the last [83].

The NRA condemns the actions of mother-led advocacy groups by drawing on a rhetorical device known as the “Hypocrite's Trap” to cast them as duplicitous in nature [84]. That is, if these groups endorse or undertake activities of their own to educate children about what to do if they encounter a gun, it invalidates their criticism of industry-funded programs. At times, the NRA frames this as evidence of their untrustworthiness, describing such acts as forms of imitation of, or theft from, the NRA program:

Imitation, so they say, is the sincerest form of flattery. But heaping condemnation on something you later steal and then try to portray as your own? Well, that’s just downright weird. And if there’s any truth to a recent blog post appearing in the New York Times, that’s what Shannon Watts – head of the Bloomberg-backed gun control group Moms Demand Action for Gun Sense in America – has done in promoting a safety course that looks suspiciously like NRA’s Eddie Eagle GunSafe^®^ program [80].

The NRA also drew on the concept of theft to frame Moms Demand Action as acting against what would be expected from mothers:

We’d like to think that mothers wouldn’t steal from us. But if we thought that, we’d be disappointed in Michael Bloomberg’s Moms Demand Action group. The Demanding Moms have more than once criticized NRA for its Eddie Eagle gun safety message […] Now the moms have claimed NRA’s message as their own. A blog post on *The New York Times*, discussing a Demanding Moms safety initiative, stated, “*That group co-founded a gun safety program that works with PTAs around the country. Children should be taught to leave the area immediately, not touch the firearm, tell an adult right away and call a parent, Ms. (Shannon) Watts said*.” Sound familiar? [85].

The same approach is used to undermine the credibility of the medical community and some media outlets whose perspectives on firearms oppose those of the gun industry and its lobby. For example, in a response to media coverage of a study published in JAMA Pediatrics that evaluated the effectiveness of a 1-min film in which a real police chief (chosen because evidence suggests figures of authority are persuasive for younger children [86]) states that guns are not toys and that children should not handle them but instead alert an adult, the NRA again framed such activities as evidence of theft:

This situation concerning the seemingly “stolen” message raises a few questions. Wouldn’t it have been easier for those at the American Medical Association to study Eddie Eagle’s rich history and fantastic results to find out what has already been proven to make children safer? And wouldn’t it have been more logical for CNN and other media covering the study to simply write an accurate headline about how Eddie Eagle has been making children safer for decades? The answer to both questions, of course, is “yes.” [87]

The article then proceeded to call into question the logic and motives underpinning the actions of the medical community and the mainstream media who are framed as lacking in honesty:

But there’s an underlying problem to such logic: Neither the organizations that claim to speak for the medical community nor the mainstream media are honest enough to admit that the people they derisively refer to as the “gun lobby” have made a huge impact on keeping America’s children safer for generations. To do so would be to admit the very people they have spent decades painting as the “bad guys” are really the “good guys.” For the mainstream media to suddenly act like they’ve discovered some secret for making kids safer around firearms is insulting, disgusting, and shameful [87].

This approach thus allows the NRA to frame the actions of their opponents as evidence that the NRA is a “good guy” and that their education program is “lifesaving”.

Finally, and despite the NRA's framings that portray the organization as an expert in child firearm safety, acting with the sole aim of protecting children from firearm injuries, the organization denounces political leaders who advocate for stronger measures based on the evidence that the ownership and accessibility of guns is what places children at risk:

Twenty U.S. Senate Democrats have issued a letter to the Government Accountability Office (GAO), asking the agency to issue a statement of best practices regarding storage of firearms in the home. The letter states that, “Death and injury by firearm is one of the most significant public health threats to young people in communities across our nation.” While the letter does not address specific practices that the GAO should endorse, it is telling that its focus is overwhelmingly on gun storage—that is, restricting all access to firearms by children—and on communicating the supposed risk of having a gun in the first place. Nowhere do the senators mention the value of campaigns designed to educate children about guns, either in the sense of the Eddie Eagle GunSafe^®^ Program or through youth shooting initiatives. When did we decide that teaching children to make responsible choices was no longer on the table [88].

The NRA's discrediting of so-called politically motivated “anti-gun zealots” with the one hand while framing themselves as an apolitical actor and therefore more trustworthy and legitimate with the other, signals the need for a more nuanced understanding of politics and critical scrutiny of such claims. The fact that firearms represent a major threat to the lives of children and adolescents in the US is a political choice and it is only through politics informed by independent and robust evidence that this threat can be addressed. The NRA obscures this reality through its framings while lobbying to maintain the *status quo*, embed unevidenced measures that place the burden on children, and undermining those who seek policy change in the interests of protecting the lives of children and young people.

## Discussion and conclusion

This article reveals how the NRA's Eddie Eagle GunSafe^®^ program and the strategies the NRA adopts to frame this as the legitimate and effective way of protecting children from firearm injuries align with the corporate political agenda of the firearm industry at the expense of children and their safety. The program presents the cause of firearm injuries and the most effective forms of prevention in ways compatible with widespread firearm ownership and thus amenable to the commercial interests of the firearm industry. Responsibility for harms is placed not with the weapons themselves, or the industry that supplies them and promotes their ownership, but with individual citizens, parents or children who may encounter these even inadvertently in their daily lives. According to the NRA it is the responsibility of even young children to avoid and mitigate the dangers created by the firearms industry and the policy regime for which they have successfully lobbied.

In addition, the NRA adopts framing and action strategies that reinforce and normalize the presence of firearms in homes and communities through the delivery of their purportedly “lifesaving” program. Firearm ownership, including by parents, is presented as normal and inevitable. They make misleading claims about the effectiveness of the program while undermining the credibility of those who question these claims and who advocate for strengthening of firearm policies to ensure the safety of children and adolescents.

Despite their efforts to frame the Eddie Eagle GunSafe^®^ program as apolitical, and to label those who advocate for alternative approaches as politically motivated “anti-gun zealots”, [81] the activities of the NRA to develop and promote the program are inherently political. While the NRA claim to be acting from a politically neutral and disinterested position, they openly lobby for what they themselves label as “pro-gun” legislation to enable the delivery of their industry-funded gun safety education intervention designed explicitly as a counter to the activities of gun control advocates. The NRA has lobbied against the adoption of laws criminalizing those who leave a firearm accessible to children by promoting gun safety education as the preferable policy approach and citing the purported effectiveness of their program [89]. Furthermore, while Eddie Eagle and The Wing Team may not ever be seen handling a gun, the program materials normalize the presence of firearms in their homes and communities, and even their mothers’ handbags.

### Contribution to the CDOH literature

The findings here are consistent with previous studies of HHIs’ and industry-funded organisations’ attempts to secure their preferred policy measures and reproduce the norms and assumptions that underpin these. For example, there is evidence that industry practices serve to problematize individuals and their supposed irresponsible or problematic behaviors (e.g. unsafe driving, problem gambling, irresponsible drinking, opioid misuse, or smoking behaviors), deflecting from the role of harmful products, marketing practices and inadequate regulation [37]. There are also notable similarities with previous analyses of industry-funded organisations and their development and promotion of public and youth-facing initiatives and programs. For example, organisations funded by the alcohol and gambling industries have been shown to make claims about the impacts of their public awareness and youth education programs and their evidence base that are unsupported by the international, independent literature on effective measures to prevent gambling and alcohol harms [40, 55, 90]. These make misleading claims about the causal relationship between their activities and public health and child safety outcomes which are mirrored here in the strategies of the NRA.

The manipulation of the concept of safety, and the vehemence with which the NRA claims to be saving lives while overtly discrediting other actors, are the more novel aspects of the practices identified here compared to other industry-funded groups. This study thus contributes to a growing body of literature demonstrating that the promotion and provision of youth education by industry actors and their affiliated organisations is a core strategy of HHIs. It functions to establish the industry’s role as part of the solution and to undermine the adoption of other measures they oppose. It serves also as a mechanism through which to promote and disseminate norms about the sale and “responsible” use of harmful products, and the key drivers of harm and safety, favourable to commercial interests and their desired maintenance of the *status quo* [38, 40, 41, 43, 55].

### Strengths, limitations and policy implications

The study has important implications for policymaking in relation to firearms and for those advocating for policy reform in the interests of protecting the safety of children and young people. While our study is limited in that we did not analyze all documents produced by the NRA related to the program since its inception, the inclusion of additional materials would add to but is unlikely to change the findings on the character and function of the framing and action strategies identified by the analysis. The claims made by the NRA that their program is “lifesaving” are particularly concerning as is their manipulation of what forms of evidence are required to establish the effectiveness of a firearm safety intervention. The NRA claims of commitment to child safety, supposedly evidenced by their involvement in the design and provision of the Eddie Eagle GunSafe^®^ program, are paradoxically contradicted by their framing and action strategies which undermine child safety by framing the effectiveness of child gun safety education in misleading ways, seeking to normalize firearm ownership, using neutralizing language, and undermining more effective measures needed to prevent child firearm injuries. Our study thus has implications for other contexts globally and child safety issues (e.g. road safety) in that it illustrates how commercial actors can frame and influence child safety problems and solutions in ways favourable to industry, while undermining the goal of keeping children safe. The approach applied in this study can be used to analyze the activities of the firearm industry in other contexts as well as generating potential insights in the activities of other commercial actors in their attempts to influence policies that impact child safety. Similarly, our findings can be used by policymakers to inform the governance of child safety and injury prevention policymaking and implementation to mitigate against undue commercial influence.

The NRA's activities in relation to their Eddie Eagle GunSafe^®^ program should be seen as distorting public understanding of what is driving firearm injuries among children, the burden of the harm, and what is needed to keep children safe. Parents, educators, and others such as police officers should not engage with materials produced by the NRA or other firearm industry-funded organisations given the body of evidence demonstrating the harmful nature of industry-funded education materials [40, 41, 43, 44]. The NRA manipulates the hierarchy of safety measures by obscuring the differences between prevention and harm reduction, and exploits widely held assumptions that educating children about harms is effective in preventing those harms [91]. Their practices need to be exposed and countered and the development of firearm policies intended to address child firearm injuries should be protected from influence by entities like the NRA.

The firearm industry and its representatives should not be seen as authoritative or informed voices on how to protect children from firearm injuries. Their expertise lies in selling and promoting firearms. Public health professionals, advocates and other stakeholders need to be aware of how their support for youth education can help to reproduce industry-favourable narratives about the effectiveness of education as a means of keeping children safe from harms, including firearm injuries. Any education program needs to be contextualized for parents, teachers and others acting to protect children and young people from firearms injuries. They should not be presented and delivered in isolation of a wider explanation regarding the likelihood of effectiveness compared to other measures that would make homes and communities free of firearms and the limited evidence base in support of the delivery of education-based programs as a form of harm prevention or reduction must be explicitly acknowledged.

Parents should be empowered to access information that is independent of the firearm industry and understand how to critique information for conflicts of interests. They have the right to be informed that having homes and communities free of firearms is the only effective way to prevent firearm injuries among all children and young people. It is deeply concerning that parents are led to believe that their children have learned what to do if they find a gun and by implication that they are somehow safe residing in a home and communities where firearms are present because of this. They remain at risk if firearms are present. Education programs, in the absence of wider firearm control measures, will only be effective at ensuring all children are protected from firearm injuries when they are able to ensure all children have learned how not to be children, who are, by definition, curious, impulsive, and risk-taking, among other characteristics that are completely natural to childhood. While firearm safety education may work for some children in some circumstances, homes and communities free of guns will work for all and the effects will be more likely to be maintained.

Finally, keeping a vulnerable group such as children safe is the responsibility of a caring society as a whole and should not be outsourced to a front group for a lethal industry with a direct conflict of interest. In this respect, as proposed in a recent analysis of UK gambling industry-funded youth education programs and industry-funded groups which misleadingly present these programs as evidence-based and evaluation-led means of safeguarding children from gambling harm, the exposure of children to industry-funded safety education programs and the functions these programs serve should be approached from a child rights perspective [40]. A child rights-based approach could help shift the debate on what has become such a divisive policy topic and should be advocated by the global health community to support progress in addressing the deeply concerning issue of child and adolescent firearm injuries in the US.

## Data Availability

All data analysed in this study are publicly available.
